# Research on the Multi-Sensor Fusion Model for Pipeline Corrosion and the Identification Method of Pitting Corrosion

**DOI:** 10.3390/s26030936

**Published:** 2026-02-01

**Authors:** Shilei Huang, Wenyang Li, Junbi Liao

**Affiliations:** 1 School of Electronic Information and Electrical Engineering, Chengdu University, Chengdu 610106, China; huangshilei@cdu.edu.cn; 2 School of Mechanical Engineering, Sichuan University, Chengdu 610065, China; liaojunbi@scu.edu.cn

**Keywords:** pipeline corrosion monitoring, field signature method, ultrasonic testing, multi-sensor fusion

## Abstract

The depth of corrosion pits is critical to the safe operation of pipelines. Conventionally, monitoring of metal pipeline corrosion has generally relied on a single technique. Among these, the ultrasonic thickness measurement method and the field signature method are widely used in pipeline monitoring systems. The ultrasonic method has high reliability and accuracy for monitoring the thickness of pipelines, but it is limited by coupling and measurement range. Additionally, the field signature method often suffers from inadequate identification of corrosion pits and lower measurement accuracy. To address these limitations, a multi-sensor fusion model is proposed to monitor corrosion in metal pipelines. The multi-sensor fusion model is constructed by alternately arranging ultrasonic sensors and field signature probes, and a dedicated fusion algorithm is designed. The integrated model leverages the complementary strengths of both techniques while mitigating their individual shortcomings. Furthermore, an artificial neural network is employed to accurately identify pitting depth, thereby resolving the challenge in discriminating corrosion pit depths. Experimental results demonstrate that the multi-sensor fusion model can overcome the inherent drawbacks associated with a single technique. Consequently, it enhances the overall reliability, measurement accuracy, and operational range of the pipeline corrosion measurement system.

## 1. Introduction

Metal pipes play a crucial role in the transportation of substances such as oil, natural gas, and chemical raw materials. As the service time of the pipeline increases, the deepening of corrosion pits on the pipeline directly affects its service life. Based on the external appearance forms of corrosion, the types of corrosion can be classified as uniform corrosion and local corrosion. Local corrosion manifests in several distinct forms, including pitting, crevice, cavitation, and galvanic corrosion [1]. Pitting corrosion is the most common type of local corrosion. Due to the varying states of pitting corrosion, monitoring of pitting corrosion is much more difficult than that of uniform corrosion [2].

**Figure 1:**
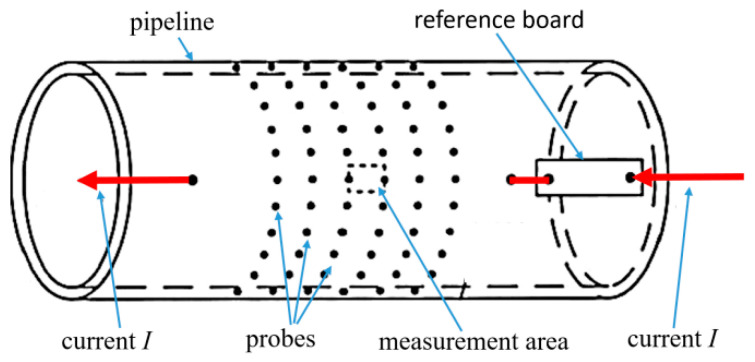
Schematic diagram of the field signature method [9].

Currently, the commonly used non-destructive monitoring techniques for pipeline corrosion mainly include the ultrasonic thickness measurement, field signature, eddy current, magnetic flux leakage, and ultrasonic guided wave methods [3,4]. Utilizing strain measurements obtained from distributed sensors, a practical sensing model was developed to assess corrosion severity and derive estimates for pit depth, mass loss, and the average corrosion rate [5]. One of the features of the measurement system is that the optical fiber is less susceptible to electromagnetic interference from buried pipelines compared to the FSM. The basic principle of the ultrasonic pulse method is to obtain the distance traveled by the ultrasonic pulse by measuring the reflection of the ultrasonic pulse within the metal pipeline wall. The ultrasonic pulse method is widely used in pipeline corrosion monitoring due to its high reliability and high measurement accuracy [6,7,8]. However, the measurement range of a single ultrasonic probe is relatively small and the cost of the probe is relatively high [4,6]. The field signature method (FSM) is an online monitoring method for metal pipeline corrosion based on potential arrays. It indirectly obtains the change in pipeline resistance by measuring the voltage variation between the external probes of the pipeline, and finally calculates the change in pipeline thickness [9]. The FSM is a non-destructive monitoring technology based on Ohm’s law. During measurement, it is necessary to weld measuring electrodes outside the metal structure to be measured and arrange them in a specific pattern to form an array (Figure 1). Each pair of measurement electrodes has a certain measurement area, which can be regarded as a resistance $R_{i,j}$. The voltage between the electrode pairs is $V_{i,j}$, and the original wall thickness of the pipe is $T_{i,j}$. When the pipeline in the measurement area is corroded, its thickness decreases and the resistance $R_{i,j}$ increases. Under the excitation of the precise constant current source *I*, the voltage $V_{i,j}$ between the electrode pairs in the corrosion area increases. By analyzing all the changes in $V_{i,j}$, the average corrosion rate inside the tube and the size of the pitting corrosion can be obtained [10,11,12].

The field signature coefficient (FC) is a parameter used to evaluate the degree of local corrosion. Its expression is:

(1) $${FC}_{i,j}=\left(\frac{V_{i,j}\left(t_{x}\right)/V_{i,j}\left(t_{0}\right)}{V_{ref}\left(t_{x}\right)/V_{ref}\left(t_{0}\right)}-1\right)\;\times \;1000$$

In the formula, $V_{i,j}\left(t_{0}\right)$ and $V_{i,j}\left(t_{x}\right)$ represent the voltages of electrode pair i,j at time $t_{0}$ and $t_{x}$, respectively; $V_{ref}\left(t_{0}\right)$ and $V_{ref}\left(t_{x}\right)$ represent the voltages of the reference electrode pair at time $t_{0}$ and $t_{x}$, respectively. To enhance clarity, a coefficient of 1000 is commonly adopted internationally, so the unit of the FC value is ppt (part per thousand). The thickness of pipeline corrosion independent of temperature and excitation current is:

(2) $${WT}_{i,j}\left(loss\right)\left(t_{x}\right)={WT}_{i,j}\left(t_{0}\right)-\frac{{WT}_{i,j}\left(t_{0}\right)\;\times \;1000}{{FC}_{i,j}+1000}$$

In the formula, ${WT}_{i,j}\left(t_{0}\right)$ represents the original thickness of the pipeline at time $t_{0}$. ${WT}_{i,j}(loss)\left(t_{x}\right)$ represents the corrosion thickness of the pipeline at time $t_{x}$. ${FC}_{i,j}$ is the field signature coefficient of the i,jth electrode pair. By calculating the wall thickness loss controlled by each pair of electrodes in the electrode array, the internal corrosion morphology of the pipeline within the layout area of the electrode array can be obtained. The field signature method has high measurement accuracy when monitoring uniform corrosion, but has lower measurement accuracy for local pitting corrosion [11,12,13]. The monitoring area is larger than that of the ultrasonic thickness measurement method, and the probe cost is lower. However, the current field signature method is not very accurate in identifying and measuring pitting corrosion [14,15,16].

Therefore, to avoid the inherent drawbacks of a single sensor, many scholars have adopted the multi-sensor fusion method to monitor the pipeline. Owojaiye et al. summarized the methods for distributing multiple sensors to monitor pipelines [17]. The article [18] utilized sensor arrays for pipeline monitoring. Papers [19,20] summarized the multi-sensor fusion technology for pipeline detection and also presented its advantages and disadvantages. Reference [21] proposed a pipeline detection method based on data fusion. A multi-source heterogeneous information fusion method with the complementary fusion of laser optical sensing and weak magnetic technologies is proposed for pipeline leakage diagnosis [22]. In the multi-sensor fusion methods, neural network methods are widely used. Articles [23,24] summarized the application of neural network methods in pipeline monitoring. Reference [25] noted that neural network methods have a relatively good effect in evaluating multiple corrosion in pipelines. Lastly, ref. [26] indicated that neural network methods can be used for the identification of small corrosion pits.

This study fully utilizes the advantages of the ultrasonic testing method (UT) and the field signature method (FSM). The interlaced array of distributed sensors and the multi-sensor fusion model are simultaneously employed to monitor pipeline corrosion. The study presented in this paper overcomes the measurement limitations of a single sensor and achieves reliable and high-precision, large-area monitoring of corrosion in active pipelines. This research accomplishes the following tasks: 1. An interlaced array distribution for pipeline corrosion monitoring based on the UT and FSM is established. A multi-sensor fusion model with high reliability, large monitoring area, and high measurement accuracy is proposed. 2. The measurement parameters for the FSM are deeply learned using artificial neural networks, and the nonlinear relationship between the depth parameters of pipeline corrosion pits and the voltage array is established. 3. The models and methods described above were tested in the field and the feasibility of the models verified.

## 2. Multi-Sensor Fusion Monitoring Model

**Figure 2:**
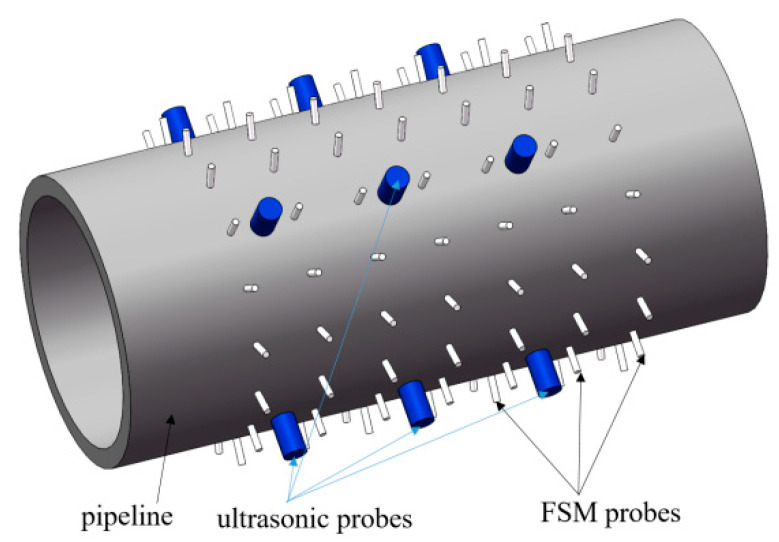
Schematic diagram of multi-sensor fusion model of FSM probes and ultrasonic probes.

The multi-sensor monitoring model of pipeline corrosion adopted in this paper consists of FSM probes and ultrasonic sensors. The probes of the FSM system are arranged in a matrix along the axial and circumferential directions of the pipeline. Additionally, the probes of the ultrasonic system are coupled to the outer surface of the pipeline and several ultrasonic probes are also arranged in a matrix (Figure 2). The ultrasonic probes are installed between the FSM probes, and they are distributed in an interlaced matrix pattern.

**Figure 3:**
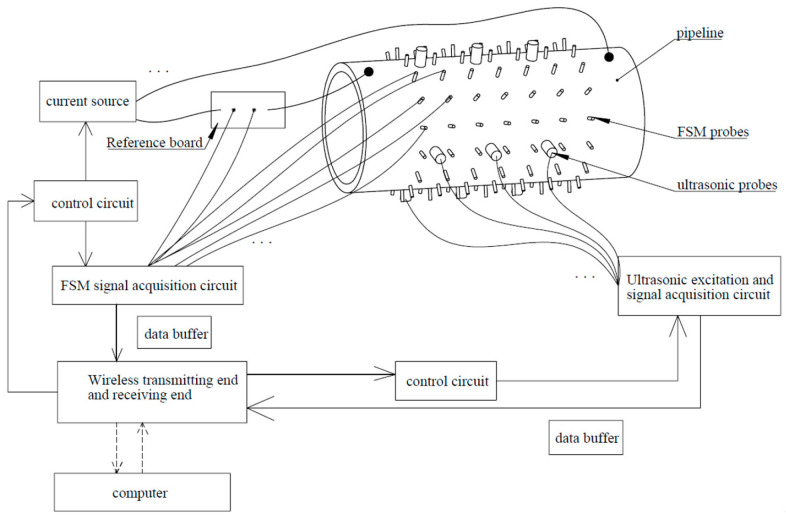
Schematic diagram of multi-sensor fusion for monitoring pipeline corrosion.

According to the model, the FSM is composed of multiple matrix-arranged probes. Each pair of probes in the axial direction corresponds to a sensor unit, and the voltage between them represents the original data layer. The voltage values between them represent the original data layer. If there is corrosion at a certain position in the pipeline, the voltage measured by the probes will change accordingly. At the same time, the ultrasonic probes between the FSM probes will also monitor the remaining thickness of the pipeline in the probe area in real time. Figure 3 is the schematic diagram of the multi-sensor monitoring model for metal pipelines. From the monitoring schematic diagram (Figure 3), the data acquisition systems of the FSM and UT are independent of each other, which prevents interference between different sensors. The FSM–UT fusion algorithm proposed in this paper is based on the stable error distribution and independence of the sensors.

**Figure 4:**
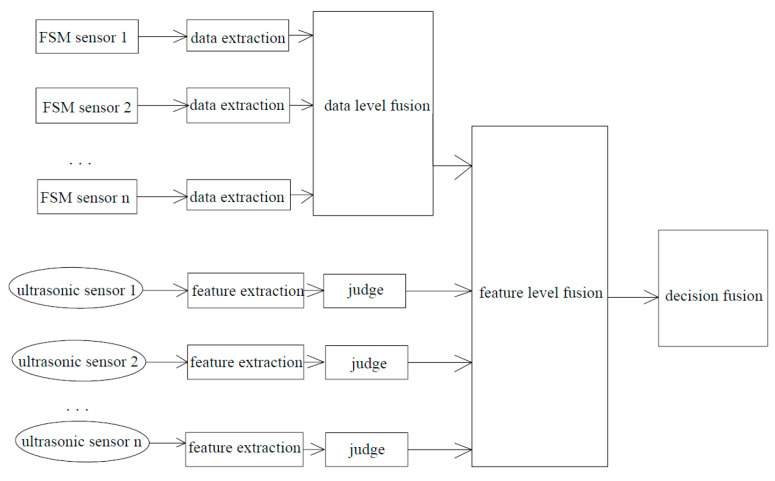
Hierarchical model of multi-sensor data fusion.

The data fusion hierarchy of the multi-sensor monitoring model is shown in Figure 4. Through data extraction, the original voltage signals collected by each pair of FSM probes are obtained. These FC values form a matrix network corresponding to the pipeline. This matrix is obtained through temperature correction and the elimination of drag effect. Through the remaining thickness algorithm, the remaining thickness matrix of the pipeline is obtained. This matrix corresponds to the actual remaining thickness of the pipeline and is the result of the fusion of the FSM system. From the echo data collected by each ultrasonic probe, the feature quantity t (time of flight) through certain methods is obtained, and then the remaining thickness of the pipeline under the probe area is obtained according to the algorithm. According to the multi-sensor distribution model, the results obtained by ultrasonic probes correspond to those of FSM probes. The remaining thickness data obtained by the two systems at the same position are explained and conclusions are drawn at the decision fusion layer.

The monitoring principle of the UT is a relatively feasible and widely recognized non-destructive monitoring technology. The UT provides accurate measurement results during uniform corrosion. However, the coverage area of the UT is small (only the area covered by the probe). Therefore, the monitoring area of the UT is relatively small. The FSM monitoring system, in principle, makes probes and the pipeline itself as sensor units, and its coverage area is large (the probe coverage area is the entire monitoring area). However, the FSM monitoring system measures the remaining thickness of the pipeline indirectly, and its accuracy is lower than that of the UT. Especially for local small corrosion pits, the accuracy is the lowest, and there is a certain temperature drift. The credible probability of the measurement results is lower than that of ultrasound, so they complement each other. Generally, for uniform corrosion, the accuracy of the FSM system is 0.5% of the wall thickness [9,11]. For example, when the original thickness is 10 mm, the accuracy of the FSM is 0.05 mm, while the ultrasonic thickness measurement accuracy is generally 0.01 mm [5,6]. Therefore, the measurement values of a single area can be fused based on the sensor accuracy.

The fusion algorithm employed in this study is based on the weighted average fusion algorithm [27,28]. Data from different sensors are linearly combined. Different sensors have different reliability and accuracy, so their weight coefficients are different. Determining the weight coefficients based on the characteristics of the sensors is a relatively mature method. Sensors with higher accuracy and greater reliability should have larger weight coefficients, while sensors with lower accuracy and poorer reliability should have smaller weight coefficients. This study determines the weight coefficients based on the measurement accuracy of the FSM and UT. The credibility and accuracy of the UT are high, and the thickness $d_{i}$ can be obtained using the weighted average Formula (3) within the small coverage area.

(3) $$d_{i}=W_{i}h_{i}+W_{j}H_{j}$$

In the formula, W represents the weight, and its value is equal to the proportion of the measurement accuracy of the corresponding sensor. $W_{i}$ is expressed as:

(4) $${\;W}_{i}=1-\frac{{∆h}_{i}}{{∆h}_{i}+{∆H}_{j}}$$

(5) $$W_{j}=1-\frac{{∆H}_{j}}{{∆h}_{i}+{∆H}_{j}}$$

Among them, ${∆h}_{i}$ represents the measurement accuracy of the sensor corresponding to the measured value $h_{i}$; ${∆H}_{j}$ represents the measurement accuracy of the sensor corresponding to the measured value $H_{j}$. For the above typical system, the weight of the FSM measurement value is 0.17, and the weight of the ultrasonic measurement value is 0.83. The thickness $d_{3}$ of the third monitoring unit is expressed as:

$$d_{3}=0.17h_{3}+0.83H_{3}$$

**Figure 5:**
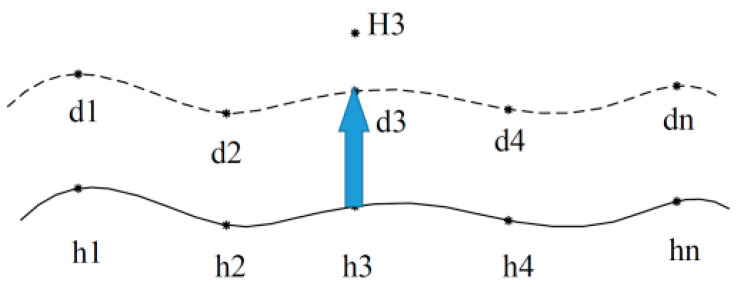
Schematic diagram of the multi-sensor fusion algorithm.

Although the coverage area of the ultrasonic monitoring system is rather limited, the high measurement accuracy of ultrasonic waves can be utilized to correct the monitoring results from the large-scale FSM system. Figure 5 illustrates the correction principle.

Based on the interlaced array distribution of the sensors, the thickness result $d_{3}$ under the monitoring area of the ultrasonic probe is first obtained (the monitoring results $h_{3}$ and $H_{3}$ of the same unit area are fused into $d_{3}$ in the feature fusion layer). The corresponding FSM monitoring result for this unit area is $h_{3}$. Therefore, the correction value is ${∆d=d}_{3}{-h}_{3}$. Around these probes, there are other probe results (the measured area is larger), which are $\left\{h_{1},h_{2}{,h}_{3}{,h}_{4},h_{5},\ldots d_{i}\right\}$. The other results $\left\{h_{1},h_{2}{,h}_{4},h_{5},\ldots d_{i}\right\}$ measured by the FSM system are adjusted by the correction value ∆d. Through fusion, the new result $d_{1i}=\left\{d_{1},d_{2},d_{3}{,d}_{4},d_{5},\ldots d_{i}\right\}$ is obtained, from which the final pipe thickness matrix D is obtained. Through the data of this matrix, a three-dimensional graph of the remaining pipe wall thickness can be drawn.

(6) $$D=\left[\begin{array}{ccc} d_{11} & \ldots & d_{1j}\\ \vdots & \ddots & \vdots \\ d_{i1} & \ldots & d_{ij} \end{array}\right]$$

This method yields results that fully take into account the measurement values from multiple sensors, reasonably integrate the measurement results of the sensors, and have certain practical value.

## 3. Identification of Pitting Depth Based on Neural Networks

**Figure 6:**
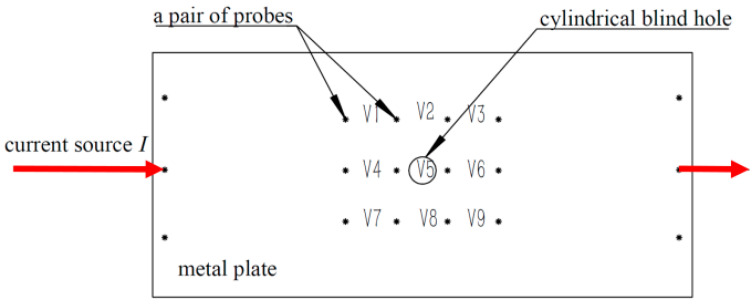
Experiment on the unreliability of a single FC value.

According to the monitoring principle of the FSM, each pair of probes and the corresponding pipeline form a sensor unit. The occurrence of corrosion affects the distribution of the current field in the FSM system. When these probes are arranged in a matrix pattern, a voltage data matrix is obtained. Therefore, the FSM is a multi-sensor system. However, a single FC value is affected by the drag effect, and there is a problem of unreliable measurement [13,14]. To better explain this phenomenon, the following experiments are described. The metal plate used in the experiments is 45 steel, with a thickness of 15 mm, and the length and width dimensions are 180 × 350 mm. The constant current source I is 30 A, and the spacing between all pairs of probes is 30 mm (Figure 6). The cylindrical blind holes are located between each pair of probes. There are a total of nine pairs of probe voltage data, which form the voltage matrix $\left\{V_{1},V_{2},V_{3},\ldots V_{9}\right\}$. From this, the FC data are obtained as $\left\{x_{1},x_{2},x_{3},\ldots x_{9}\right\}$.

From Table 1, it can be seen that the FC values of cylindrical pitting with different diameters and depths may be the same or similar, which is one of the reasons for the failure identification of pitting depth [14,15]. This problem seriously affects the identification of pitting depth by the FSM monitoring system.

**Figure 7:**
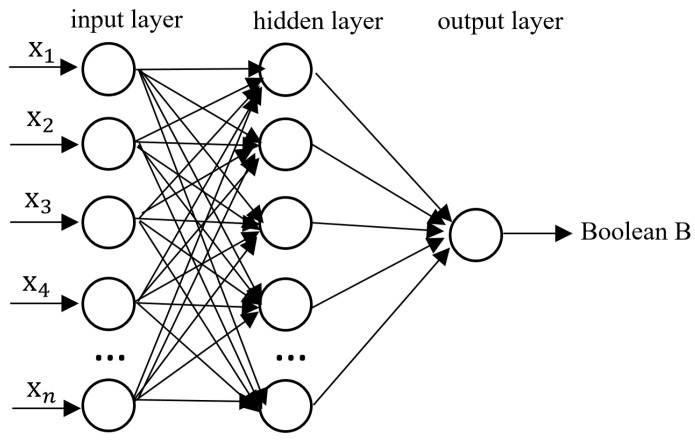
Graph of the artificial neural network model for identifying the depth of pitting corrosion.

The literature indicates that the FC value of a corrosion pit with a large corrosion area but a small corrosion depth may be the same as that of a corrosion pit with a small corrosion area but a large corrosion depth [12]. Therefore, it is difficult to estimate the pitting depth with a single FC value, while a multi-point FC value matrix may better discriminate the pitting depth. The corrosion changes are correlated with the probe voltage, which will cause different variations in the FSM probe voltage values around the pitting corrosion area. During the monitoring process, it is difficult to accurately calculate the size due to pitting corrosion. Therefore, the identification of the pitting depth is of great significance for predicting the dangerous points of the pipeline. The change in FC is expressed as the input matrix $\left\{x_{1},x_{2},x_{3},\ldots x_{n}\right\}$, and the output is a Boolean quantity B (Figure 7). The output Boolean quantity of 1 indicates that the pitting depth is greater than or equal to twice the pipe wall thickness. The Boolean quantity of 0 represents a pitting depth that is less than twice the pipe wall thickness. Therefore, this classification method is adopted in this study to distinguish between deep and shallow pits in order to improve the reliability of the FSM technology. The binary classification method is employed to determine whether the depth of the pit exceeded half of the pipe wall thickness. This was mainly to compensate for the shortcomings of the FSM technology, rather than a standard method. Formula (1) defines FC. Vi represents the voltage between the FSM probes (along the direction of the current). The vector matrix $\left\{x_{1},x_{2},x_{3},\ldots x_{n}\right\}$ is the matrix form of the FC values, representing all the FC values within a probe array.

The changes in the cylindrical pitting radius and depth $\left\{r,h\right\}$ lead to the changes in the matrix $\left\{x_{1},x_{2},x_{3},\ldots x_{n}\right\}$. Identifying the non-linear relationship between them can effectively determine whether the pitting depth is too large. Artificial neural networks, as a commonly used algorithm for multi-sensor fusion, have many advantages. In this study, its non-linear processing capability is utilized to resolve this non-linear problem.

## 4. Experiments and Discussion

Using artificial defects to validate new models instead of real defects is a common approach. Due to the limitations of the FSM for detecting clustered pitting and considering that some researchers regard corrosion smaller than the area of a pair of probes as small pitting corrosion [12,13], this paper only discusses the situation of single-point corrosion within the range of a pair of probes of the FSM. Another reason for choosing mechanical processing defects as the verification dataset instead of using actual corrosion is to eliminate the influence of other factors. This experiment aims to verify whether the FC dataset can distinguish the depth of single-point pitting corrosion, and the main purpose is to compensate for the shortcomings in the FSM technology.

The simulation data were used for the training set, while the experimental data were used for the validation data in the model. The data used for training the artificial neural network were grouped in sets of nine FCs; that is, the input was $\left\{x_{1},x_{2},x_{3},\ldots x_{9}\right\}$. There were a total of 126 data groups for training, corresponding to the radii of cylindrical pitting (r = 0.5, 1, 3, 5, 7, 9, 11, 13, 15 mm) and depths (h = 1, 2, 3, 4, 5, 6, 7, 8, 9, 10, 11, 12, 13, 14 mm). After training, the neural network model was obtained. The artificial neural network used in the experiment was the classic artificial neural network. They were, respectively, the BP neural network, the feedback neural network and the radial-basis neural network. The BP neural network adopted in this study has three layers: the input layer, the hidden layer, and the output layer. The number of neurons in the input layer is nine. The number of nodes in the hidden layer is also nine. The activation functions of the hidden layer and the output layer are the sigmoid function and the linear function, respectively. The maximum training times are 10,000 (epochs). The minimum mean square error is $1\times 10^{-8}$. The feedback neural network (Elman neural network) adopted in this study has four layers: the input layer, the hidden layer, the concatenation layer, and the output layer. The number of neurons in the input layer is nine. The number of neurons in the hidden layer is thirty. The activation functions of the hidden layer and the output layer are the hyperbolic tangent activation function and the sigmoid function, respectively. The maximum training times are 10,000 (epochs). The minimum mean square error is 0.01. The radial-basis neural network adopted in this study is a probabilistic neural network (PNN). Its neural network has three layers: the input layer, the hidden layer, and the output layer. The activation function is the Gaussian function. The expansion coefficient is 0.02.

To verify the identification capability of the network, experiments were conducted using six sets of data with radii of 3.5 mm, 5.5 mm, and 8.5 mm, corresponding to depths of 2, 4, 6, 8, 10, and 12 mm (Table 2 and Table 3).

The experiment shows that the method based on neural networks used in this study has an accuracy rate of more than 90% for the identification of pitting depth. The classification is accurate at the deep pit and the shallow pit, indicating that the data fusion algorithm combining the BP neural network and the feedback neural network is applicable for the identification of cylindrical pitting depth, and has considerable practicality.

Table 4 presents the results of using the radial-basis neural network to identify the depth of cylindrical pitting. The identification error of the radial-basis neural network is larger than that of the previous two neural network methods. The experiment revealed that the radial-basis neural network method encountered problems when integrating the data from the FSM probe for the identification of pitting depth. Therefore, these issues should be addressed at the decision-level fusion.

We installed and deployed two sets of the proposed multi-sensor fusion models in the Puguang oil field in southwestern China. All the monitoring data are sequentially transmitted back to the data analysis center (the computer system in the monitoring room) via the wireless transmitting end. The monitoring interval is set at one hour, which ensures the long-term continuity of the data. A large amount of data enters the database and is stored for the system to retrieve and analyze. Table 5 presents the data on the remaining wall thickness of the pipeline in this monitoring model. The pipeline is a commonly used metal pipeline for oil and gas transportation, with a nominal thickness of 10 mm and an inner diameter of 220 mm. Nine FSM probes are axially arranged along the pipeline, and fourteen groups of FSM probes are circumferentially arranged, forming a multi-sensor matrix of 14 × 9. The distribution of the FSM probes has a certain impact on the resolution of the measurement. The small probe spacing makes the measurement more sensitive. However, the probe spacing is too small, resulting in a relatively low voltage between the probes (with a relatively small FC value). The measurement error for smaller voltage values will increase. A large number of papers select a spacing of the FSM probes at usually two to five times the pipeline thickness, and the current intensity is 30 to 40 amperes [10,12,29]. The low current results in a relatively small voltage for the FSM probe, leading to an increased measurement error. Excessive current may cause overheating or affect the explosion-proof safety. The UT probe can only monitor the corrosion condition of the pipeline within its own area. Therefore, from the perspective of spatial layout, the arrangement of UT probes depends on the FSM probes. The probe spacing selected for this experiment is 30 mm. The size of the probe matrix is determined according to the scope of pipeline monitoring. Theoretically, the number of probe points of the FSM can be of any size. However, considering the cost and the selection of the monitoring range, the probe matrix size used for the oil field selected in this study is 14 × 9. The monitoring technology proposed in this article is mainly used for buried pipelines with encapsulated sealing layers. Therefore, probes are almost completely isolated from the soil. However, numerous studies have shown that temperature has a significant impact on the FSM and UT [10,12,13,30,31]. Temperature variations cause drift in the FSM, and ultrasonic sensors require temperature correction for the sound velocity. The ultrasonic monitoring data presented in this article had already undergone sound velocity correction.

Two ultrasonic probes are axially arranged on the pipeline and are staggered with the FSM probes. After the installation of the multi sensor monitoring model, it is sealed with glue and buried. The monitoring data are transmitted to the data analysis center, where the final calculation of the remaining wall thickness is carried out at the thickness decision-level fusion. The bolded data represent the remaining wall thickness data measured by different sensors at the same monitoring point of the pipeline. There is a significant difference between the monitoring results of the FSM system and the UT system. After fusion, the remaining wall thickness in the measurement area of the pipeline is obtained, and the credibility of the measurement value is enhanced. Additionally, the multi-sensor fusion monitoring model also reduces the impact of the FSM probe failure.

**Figure 8:**
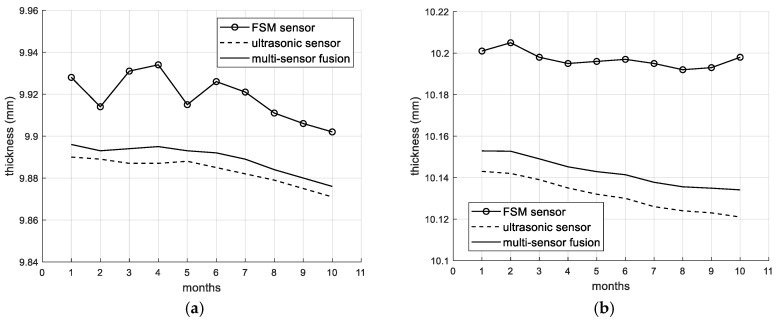
Comparison of the stability of multi-sensor fusion monitoring models. (**a**) The detection curve of monitoring point 1-2. (**b**) The detection curve of monitoring point 1-6.

**Figure 9:**
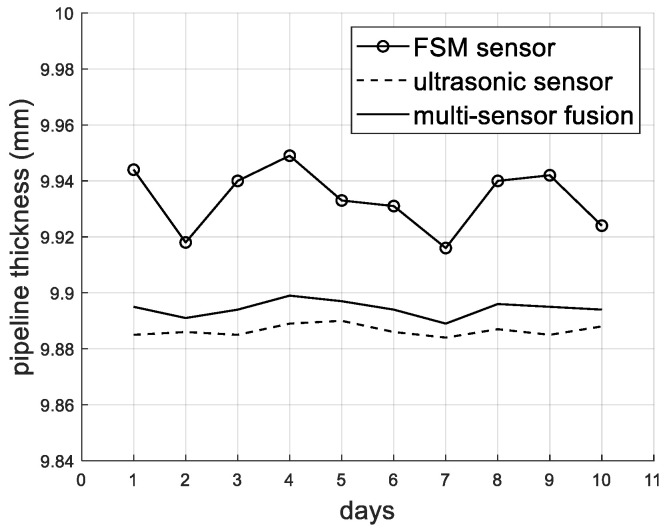
Curves of the pipeline thickness measured by different methods.

Figure 8 shows the curve of pipe thickness change after 10 months of monitoring. Although the overall trend in the FSM monitoring values is consistent with that of the ultrasonic monitoring values, the drift of the FSM is relatively large (with larger fluctuations as well). The ultrasonic sensor used in this study is equipped with a thermocouple temperature sensor to collect the temperature data near the ultrasonic probe. The ultrasonic sound velocity is corrected according to the corresponding temperature (temperature–sound velocity relationship curve), which reduces the influence of temperature on the ultrasonic sensor. Then, based on the fusion model proposed in this article, the FSM data are corrected. Table 6 presents the statistical results of the pipeline thickness at the same point monitored by different methods over a period of 10 days. Figure 9 shows the curves representing the changes in pipeline thickness data monitored by different methods. It can be seen that the FSM has a large drift and a large standard deviation. The standard deviations of the UT and fusion model methods are relatively smaller. The statistical results show that the fusion model method proposed in this paper has better stability and smaller drift compared to the single FSM.

After using the model proposed in this paper for multi-sensor data fusion, the fluctuations in the fusion results are significantly reduced, and the monitoring results become more stable and reliable. The existing pipeline corrosion monitoring technologies mainly employ a single type of sensor. Especially for the FSM technology, there is currently no effective method to completely eliminate drift [12,13,14,15]. This paper innovatively establishes the sensor layout of FSM and UT, and enhances the reliability and measurement accuracy of the FSM monitoring system by the fusion model. The multi-sensor fusion monitoring model, due to long-term fixed monitoring (such as underground installation), requires the system to have good fault-tolerance and stability. If a pair of FSM probes or an ultrasonic probe in the monitoring system fails in coupling or installation, the data from other sensors will compensate for the missing or erroneous part. This approach enhances the stability of the monitoring system, providing the necessary conditions for long-term monitoring.

## 5. Conclusions

The main innovation of this study is its proposal for a multi-sensor pipeline corrosion monitoring model that combines FSM and UT technology. The principle and interlaced array distribution structure of the model are described, along with the corresponding fusion algorithm. The final residual thickness of the metal pipeline is derived from the model that fuses FSM and UT data. Furthermore, to reduce the misjudgment of the FSM technology for deep and shallow pits, artificial neural networks are also employed to identify the depth of pitting corrosion. Experimental results demonstrate that this method can effectively distinguish shallow and deep pits, preventing the misjudgment of a single FC value. The proposed fusion model addresses three major limitations: it improves the measurement accuracy of FSM systems, expands the effective monitoring range of ultrasonic sensing, and reduces temperature-induced drift. Compared to a single technique, the monitoring model has improved fault-tolerant ability, stability and credibility of monitoring results.

## Figures and Tables

**Table 1:** The values of FC corresponding to different cylindrical pitting corrosion.

Radius r (mm)	1 mm	3 mm	5 mm	7 mm	9 mm	The Voltage of FSM Probes
Depth h (mm)	0.02	0.16	0.39	0.73	1.18	V1
2 mm	0.11	0.87	2.25	4.20	6.72	V2
	0.02	0.16	0.39	0.73	1.18	V3
	−0.11	−0.82	−2.01	−3.48	−4.99	V4
	**0.42**	**3.22**	**8.04**	**14.49**	**22.05**	**V5**
	−0.11	−0.82	−2.01	−3.48	−4.99	V6
	0.02	0.16	0.39	0.73	1.18	V7
	0.11	0.89	2.25	4.20	6.72	V8
	0.02	0.16	0.39	0.73	1.18	V9
4 mm	0.05	0.34	0.87	1.65	2.63	V1
	0.25	1.96	5.05	9.44	15.04	V2
	0.05	0.34	0.87	1.65	2.63	V3
	−0.23	−1.88	−4.63	−8.02	−11.46	V4
	**0.92**	**7.28**	**18.35**	**32.97 **	**49.93**	**V5**
	−0.23	−1.88	−4.65	−8.02	−11.46	V6
	0.05	0.34	0.87	1.65	2.63	V7
	0.25	1.96	5.05	9.44	15.04	V8
	0.05	0.34	0.87	1.65	2.63	V9
6 mm	0.06	0.53	1.40	2.64	4.26	V1
	0.37	3.11	8.12	15.25	24.40	V2
	0.06	0.53	1.40	2.64	4.26	V3
	−0.39	−3.14	−7.84	−13.72	−19.73	V4
	**1.45**	**11.85**	**30.25 **	**54.75**	**83.27**	**V5**
	−0.39	−3.14	−7.85	−13.72	−19.75	V6
	0.06	0.53	1.40	2.64	4.26	V7
	0.37	3.11	8.12	15.25	24.40	V8
	0.06	0.53	1.40	2.64	4.26	V9

**Table 2:** The identification results based on BP neural network.

Depth h (mm)	2	4	6	8	10	12
Boolean B (r = 3.5 mm)	0	0	0	1	1	1
Boolean B (r = 5.5 mm)	0	0	0	1	1	1
Boolean B (r = 8.5 mm)	0	0	0	1	1	1
Judgment result (X indicates a misjudgment)	-	-	-	-	-	-

**Table 3:** The identification results based on feedback neural network.

Depth h( mm)	2	4	6	8	10	12
Boolean B (r = 3.5 mm)	0	0	0	**0**	1	1
Boolean B (r = 5.5 mm)	0	0	0	1	1	1
Boolean B (r = 8.5 mm)	0	0	0	1	1	1
Judgment result (X indicates a misjudgment)	-	-	-	X	-	-

**Table 4:** The identification results based on the radial-basis neural network.

Depth h (mm)	2	4	6	8	10	12
Boolean B (r = 3.5 mm)	0	0	0	1	1	1
Boolean B (r = 5.5 mm)	0	**1**	**1**	1	1	1
Boolean B (r = 8.5 mm)	0	0	**1**	**0**	1	1
Judgment result (X indicates a misjudgment)	-	X	X	X	-	-

**Table 5:** Experimental data of the multi-sensor fusion model for monitoring the remaining thickness of pipelines (unit: mm).

Monitoring Time	Identification Number of the Monitoring Point	1-1	1-2	1-3	1-4	1-5	1-6	1-7	1-8
1 October 2024	FSM data	9.532	9.921	9.674	9.817	10.315	10.206	9.985	9.773
	UT data		9.892				10.148		
	result of multi-sensor fusion	9.508	**9.897**	9.650	9.793	10.267	**10.158**	9.937	9.725
1 April 2025	FSM data	9.524	9.934	9.665	9.815	10.337	10.195	9.969	9.748
	UT data		9.887				10.135		
	result of multi-sensor fusion	9.485	9.895	9.626	9.776	10.287	10.145	9.919	9.698
1 October 2025	FSM data	9.501	9.902	9.660	9.808	10.283	10.198	9.983	9.761
	UT data		9.871				10.121		
	result of multi-sensor fusion	9.475	**9.876**	9.634	9.782	10.219	**10.134**	9.919	9.697

**Table 6:** Statistical results of pipeline thickness data obtained through different monitoring methods (unit: mm).

	FSM	UT	Fusion Model
Standard deviation (probe 1-2)	0.0113	0.002	0.0028
Percentage reduction in standard deviation (Compared to FSM)	-	82.3%	75.2%
Standard deviation (probe 1-6)	0.0091	0.0019	0.0026
Percentage reduction in standard deviation (Compared to FSM)	-	79.1%	71.4%

## Data Availability

The original contributions presented in this study are included in the article. Further inquiries can be directed to the corresponding author.
